# Socioeconomic Deprivation, Genetic Risk, and Incident Dementia

**DOI:** 10.1016/j.amepre.2023.01.012

**Published:** 2023-05

**Authors:** Matthias Klee, Anja K. Leist, Michele Veldsman, Janice M. Ranson, David J. Llewellyn

**Affiliations:** 1 Institute for Research on Socio-Economic Inequality, Department of Social Sciences, University of Luxembourg, Esch-sur-Alzette, Luxembourg; 2 The Deep Dementia Phenotyping Network, Exeter, United Kingdom; 3 Wellcome Centre for Integrative Neuroimaging, Department of Experimental Psychology, University of Oxford, Oxford, United Kingdom; 4 College of Medicine and Health, University of Exeter, Exeter, United Kingdom; 5 The Alan Turing Institute, London, United Kingdom

## Abstract

**Introduction:**

Socioeconomic factors and genetic predisposition are established risk factors for dementia. It remains unclear whether associations of socioeconomic deprivation with dementia incidence are modified by genetic risk.

**Methods:**

Participants in the UK Biobank aged ≥60 years and of European ancestry without dementia at baseline (2006–2010) were eligible for the analysis, with the main exposures area-level deprivation based on the Townsend Deprivation Index and individual-level socioeconomic deprivation based on car and home ownership, housing type and income, and polygenic risk of dementia. Dementia was ascertained in hospital and death records. Analysis was conducted in 2021.

**Results:**

In this cohort study, 196,368 participants (mean [SD] age=64.1 [2.9] years, 52.7% female) were followed up for 1,545,316 person-years (median [IQR] follow-up=8.0 [7.4–8.6] years). In high genetic risk and high area-level deprivation, 1.71% (95% CI=1.44, 2.01) developed dementia compared with 0.56% (95% CI=0.48, 0.65) in low genetic risk and low-to-moderate area-level deprivation (hazard ratio=2.31; 95% CI=1.84, 2.91). In high genetic risk and high individual-level deprivation, 1.78% (95% CI=1.50, 2.09) developed dementia compared with 0.31% (95% CI=0.20, 0.45) in low genetic risk and low individual-level deprivation (hazard ratio=4.06; 95% CI=2.63, 6.26). There was no significant interaction between genetic risk and area-level (*p=*0.77) or individual-level (*p=*0.07) deprivation. An imaging substudy including 11,083 participants found a greater burden of white matter hyperintensities associated with higher socioeconomic deprivation.

**Conclusions:**

Individual-level and area-level socioeconomic deprivation were associated with increased dementia risk. Dementia prevention interventions may be particularly effective if targeted to households and areas with fewer socioeconomic resources, regardless of genetic vulnerability.

## INTRODUCTION

The risk of Alzheimer disease (AD) and other subtypes of dementia is determined by multiple pathways, including genetic, environmental, and lifestyle factors.^1^ Most cases occur in older adults, and risk is linked to multiple common genetic variants, with population-attributable fractions for single nucleotide polymorphisms (SNPs) of up to 8% or 27.3% for apolipoprotein e4 allele (APOE).^2^ Many studies have therefore employed polygenic risk scores (PRSs) to quantify the genetic risk of dementia, suggesting almost 2 times higher incidence in high than in low polygenic risk.^2^, ^3^, ^4^, ^5^, ^6^

Moreover, individuals with fewer socioeconomic resources are at higher risk of dementia.^6^, ^7^, ^8^ Socioeconomic deprivation has been measured before using both individual-level indicators such as income or wealth and area-level indices such as the Townsend Deprivation Index^4^^,^^6^^,^^7^^,^^9^ that captures unemployment rates, car and home ownership, and household overcrowding.^9^ Despite lower population-attributable fractions of risk factors related to socioeconomic deprivation (air pollution, 2.3%; education, 7.1%), recent findings suggest higher importance of wealth-related than genetic risk factors.^1^^,^^10^ Whereas low area-level socioeconomic deprivation has been linked to cognitive reserve and lower rates of cognitive decline in some studies, others found that area-level deprivation was no longer significant after adjustment for individual-level wealth.^7^^,^^11^^,^^12^ This suggests that previous studies captured potentially distinct drivers of associations such as access to green space or air pollution, which are yet to be fully understood.^13^^,^^14^

Cross-sectional findings link higher area-level deprivation to AD neuropathology.^8^ Furthermore, a recent study found that higher socioeconomic deprivation, among others, is associated with higher brain age.^15^ Longitudinally, cognitive decline and accelerated degeneration in signature regions of AD, including the medial temporal lobe, were associated with higher area-level socioeconomic deprivation.^16^ In addition, links of white matter hyperintensities (WMHs) to a more rapid cognitive decline in patients with mild cognitive impairment have been established before.^17^

No study has yet investigated the interplay of said factors jointly. Consequently, net associations of area-level above individual-level socioeconomic deprivation and their potential mechanisms have not been fully elucidated. Although polygenic scores quantify a diathesis for dementia, it is yet to be examined whether genetic predisposition may exacerbate the associations of area-level and individual-level socioeconomic deprivation with incident dementia. Earlier research found interactions of polygenic risk with wealth and educational attainment and of APOE genotype with smoking.^6^^,^^18^ Findings show improved resilience to AD-related neurodegeneration, but there is also evidence suggesting more complex interactive pathways involving inflammation, which are not well understood yet.^6^^,^^19^, ^20^, ^21^ Identifying potential interaction effects is crucial because they may point to risk factors and population groups that are most effective to target in dementia risk reduction interventions.

The purpose of this study was to use data from a large population-based cohort to investigate the hypothesis that associations between individual- and area-level socioeconomic deprivation and dementia may be modified by genetic risk. Complementary to previous research, the UK Biobank study offers unique opportunities. With over 500,000 participants, analyses are well powered to detect potentially small interactions. In addition, information on genetics, imaging, and area-level and individual-level socioeconomic deprivation is provided. Finally, linkage to health records and death registries allows extensive follow-up and dementia ascertainment.^22^

## METHODS

### Study Sample

Data were provided by the UK Biobank, a population-based cohort study in the United Kingdom.^23^ Participants completed baseline assessments between 2006 and 2010 hosted in 22 centers.^23^ Of 502,536 participants, 196,368 were eligible for analysis, excluding participants aged below 60 years (*n*=285,037), of ancestry other than European or without genetic data (*n* = 20,969), with dementia at baseline (*n*=147), or who discontinued consent before the time of analysis (*n*=15). Follow-up continued until the date of first diagnosis, death, dropout, or last hospital admission. Participants without technical exclusion criteria (e.g., metal implants, discontinued consent, high movement) were reinvited for imaging between 2014 and 2020.^23^, ^24^, ^25^ A neuroimaging substudy included 11,083 eligible participants with imaging data.

### Measures

Area-level socioeconomic deprivation was assessed with the Townsend Deprivation Index, including information on employment, home ownership, car ownership, and household overcrowding, on the basis of baseline assessments and the preceding national census output areas.^9^ Area-level socioeconomic deprivation categories distinguish low-to-moderate (Quintiles 1–4) and high (Quintile 5) deprivation because exploratory analyses suggested no significant differences in the associations of Quintiles 1–4 with dementia risk ( Appendix Figure 1, available online).

Individual-level socioeconomic deprivation was based on a weighted composite score, including home (own home without mortgage, other/not disclosed) and car ownership (one or more, none/not disclosed), housing type (house/flat, other/not disclosed), and annual household income before tax (>£31,000, £18,000–30,999, <£18,000, not disclosed). For comparison, the median equivalized net household income in the United Kingdom in 2010/2011 (end of baseline) was ∼£22,000. The coefficients of a Cox proportional-hazards regression, with time-to-incident all-cause dementia as the outcome, were used to compute individual-level deprivation (Appendix Table 1, available online). The score sums the product of indicators and their regression coefficient and divides it by the total sum of coefficients. Categories distinguish low (Quintile 1), intermediate (Quintiles 2–4), and high (Quintile 5) individual-level socioeconomic deprivation. Previous research suggests systematic differences between participants disclosing socioeconomic indicators such as income and those that do not.^26^ Therefore and owing to group sizes, not disclosed information was merged with less favorable categories except for income, where it was kept as a separate category. A sensitivity analysis excluded participants who did not disclose socioeconomic information, yielding similar results (Appendix Table 1, available online).

The PRS quantifies AD and dementia risk.^4^ Polygenic risk was operationalized as the z-standardized weighted sum of the number of prevalent alleles at each AD-related SNP, including APOE genotype. Weights are based on their association with AD determined in a meta-analysis of genome-wide association studies of individuals of European ancestry.^2^ Therefore, analyses were restricted to participants of self-identified European ancestry (British, Irish, other White). In total 249,273 SNPs met the *p*-value threshold for inclusion (*p*<0.50).^4^ Polygenic risk groups distinguish low (Quintile 1), intermediate (Quintiles 2–4), and high (Quintile 5) risk.

Participants’ all-cause dementia status was derived from hospital inpatient data (England: Hospital Episode Statistics; Scotland: Scottish Morbidity Record; and Wales: Patient Episode Database) and death records (England and Wales: National Health Service Digital; Scotland: Information and Statistics Division); coding International Classification of Diseases, Ninth Revision/ICD-10 denoted primary/secondary dementia diagnosis or dementia-related cause of death.^27^ International Classification of Diseases codes are presented in the supplementary material of a previous publication.^4^ Previous research suggests the high validity of this protocol, balancing a positive predictive value of 84.5% with reasonable case ascertainment.^22^ The 6 imaging-derived phenotypes (WMH, whole brain, gray matter, white matter, left and right hippocampal volume) were generated by an image-processing pipeline developed and run on behalf of UK Biobank.^24^^,^^28^, ^29^, ^30^, ^31^, ^32^, ^33^, ^34^

All models were adjusted for baseline characteristics, including age in years, education (high: college/university degree; medium: higher secondary; low: lower secondary; other: degrees not covered in response options/nonresponse), sex, marital status (living with husband/wife/partner, joint category other/not disclosed), ancestry (20 first principal components), and in-sample third-degree relatedness.^35^ Models including PRS were additionally adjusted for the number of alleles included during computation. Potential mediators presence of depressive symptoms in the last 2 weeks and a healthy-lifestyle score (favorable, intermediate, unfavorable) were included in the main analysis.^4^^,^^36^

### Statistical Analysis

Missing data were assumed missing at random and addressed using multiple imputation by chained equations with 5 imputations.^37^ The imputation procedure employed recursive partitioning, which is beneficial in the presence of nonlinear relations.^38^ Dementia incidence, survival times, variables relating to genetic risk or imaging, age, sex, and housing type were complete in eligible participants (Appendix Figure 2, available online).

Cox proportional-hazards regressions were applied to investigate the relationship of individual-level and area-level socioeconomic deprivation with time-to-incident all-cause dementia. Time at risk of dementia was modeled from baseline until diagnosis, loss to follow-up, death, or end of hospital admissions (England: March 31, 2017; Wales: February 29, 2016; Scotland: October 31, 2016). Main exposures were introduced stepwise to confirm main associations. Interaction terms between socioeconomic deprivation and polygenic risk were tested to investigate moderation. The assumption of proportional hazards was confirmed using Schoenfeld residuals (*p=*0.71 in the first imputed data set).^39^

For the main analysis, socioeconomic deprivation categories were combined with polygenic risk groups, with low genetic risk and lower socioeconomic deprivation as reference categories, to investigate variation in the associations of deprivation with dementia incidence for different levels of genetic risk. Absolute risk was calculated as the percentage of cases on the basis of the first imputed data set. Incidence rates per 1,000 person-years were calculated accordingly.

For the exploratory imaging substudy, potential imaging-related confounders were entered in the multivariable linear regressions as predictors for imaging-derived phenotypes, including site-specific derivatives capturing (squared) age, sex, age‒sex interactions, head size, (squared) days since scanner start-up, and 2 dummy variables coding site. In a second step, scaled residuals were used as dependent variables in multivariable linear regressions, including main exposures, covariates, and inverse probability weights on the basis of logistic regression models with selection into the imaging subsample as the dependent variable.^40^, ^41^, ^42^ WMH burden was log transformed.

Sensitivity analyses comprised replication in complete-case data and subsamples stratified by polygenic risk and sex. For the imaging substudy, a less conservative set of potential imaging-related confounders, including age, sex, age‒sex interactions, head size, and site, was applied.^41^

Results were pooled across 5 imputed data sets according to Rubin's rules.^43^ Significance was assessed 2-sided with *p*<0.05. Analyses were performed in R, Version 4.0.3.^43^, ^44^, ^45^ Analysis code will be made available on the GitHub page of the first author (https://github.com/makleelux).

The UK Biobank study received approval from the North West Multi-centre Research Ethics Committee, the National Information Governance Board for Health & Social Care, and the Community Health Index Advisory Group. All participants signed informed consent at baseline.

## RESULTS

In total, 196,368 (mean [SD] age=64.1 [2.9] years) participants (52.7% female) were followed up for 1,545,316 person-years (median [IQR] follow-up=8.0 [7.4–8.6] years). During follow-up, 1,769 participants developed dementia (Table 1^4,9,35^). In complete-case data, the median age at dementia diagnosis was 72.0 years for low-to-moderate and 71.7 years for high area-level deprivation and 71.6 years for low, 72.0 years for intermediate, and 71.7 years for high individual-level deprivation.

**Table 1:** Baseline Characteristics of Participants

Characteristics	Total *n* (%)^a^	Total *n* (%)^a^
	Incident dementia (*n*=1,769)	No incident dementia (*n*=194,599)
Age, years, mean (SD)	65.8 (2.7)	64.1 (2.8)
Sex		
Female	790 (44.7)	102,644 (52.8)
Male	979 (55.3)	91,955 (47.2)
Education^b^^,^^c^		
High	317 (17.9)	49,493 (25.4)
Medium	472 (26.7)	59,160 (30.4)
Low	255 (14.4)	30,939 (15.9)
Other^d^	725 (41.0)	55,007 (28.3)
Married or in a relationship^c^	1,586 (89.7)	179,256 (92.1)
Depressive symptoms in last 2 weeks^c^	411 (23.2)	32,942 (16.9)
Healthy lifestyle^c^^,^^e^		
5 (favorable)	251 (14.2)	39,022 (20.1)
2–4 (intermediate)	1,049 (59.3)	116,772 (60.0)
1 (unfavorable)	469 (26.5)	38,805 (19.9)
Individual-level socioeconomic deprivation^c^^,^^e^^,^^f^		
1 (low)	174 (9.8)	39,100 (20.1)
2–4 (intermediate)	1,037 (58.6)	116,784 (60.0)
5 (high)	558 (31.6)	38,715 (19.9)
Area-level socioeconomic deprivation^c^^,^^e^^,^^g^		
1–4 (low-to-moderate)	1,266 (71.6)	155,829 (80.1)
5 (high)	503 (28.4)	38,770 (19.9)
Genetic Risk Group^e^^,^^h^		
1 (low)	247 (14.0)	39,027 (20.1)
2–4 (intermediate)	1,038 (58.7)	116,783 (60.0)
5 (high genetic)	484 (27.4)	38,789 (19.9)

ISCED, International Standard Classification of Education; UNESCO, United Nations Educational, Scientific and Cultural Organization.

a Percentages may not sum to 100 because of rounding.

b Education was grouped on the basis of the UNESCO ISCED 2011^35^ classification system.

c Missing values have been imputed. Reported values are averaged across 5 imputed data sets.

d The response level other summarized options prefer not to answer and none of the above.

e Categories based on continuous scores. Numbers indicate quintiles from lowest (1) to highest (5).

f Individual-level socioeconomic deprivation summarizes information on home and car ownership, housing type, and income.

g Area-level socioeconomic deprivation based on the Townsend Deprivation Index.^9^

h Genetic risk based on a polygenic risk score for dementia.^4^

Dementia risk was higher in participants living in areas with fewer socioeconomic resources. Of participants in high area-level deprivation, 1.28% developed dementia (95% CI=1.17, 1.40) (Appendix Table 2, available online) versus 0.81% (95% CI=0.76, 0.85) in low-to-moderate area-level deprivation (adjusted hazard ratio [HR]=1.47, 95% CI=1.32, 1.63) (Table 2). Inclusion of genetic risk resulted in an adjusted HR of 1.47 (95% CI=1.32, 1.64), indicating that area-level deprivation is independent of genetic risk. Additional inclusion of individual-level deprivation resulted in an adjusted HR of 1.28 (95% CI=1.14, 1.43), suggesting that the association between area-level deprivation and dementia risk is partially accounted for by individual-level deprivation.

**Table 2:** Risk of Incident Dementia According to Area-Level Socioeconomic Deprivation

Area-level socioeconomic deprivation^a^	Model 1^b^	Model 1^b^	Model 2^c^	Model 2^c^	Model 3^d^	Model 3^d^
	Low-to-moderate (*n*=157,095)	High (*n*=39,273)	Low-to-moderate (*n*=157,095)	High (*n*=39,273)	Low-to-moderate (*n*=157,095)	High (*n*=39,273)
Number of dementia cases/person-years^a^	1,266/1,240,516	503/304,799	1,266/1,240,516	503/304,799	1,266/1,240,516	503/304,799
HR (95% CI)	1 (ref)	1.47 (1.32, 1.63)	1 (ref)	1.47 (1.32, 1.64)	1 (ref)	1.28 (1.14, 1.43)
*p*-value		**<0.001**		**<0.001**		**<0.001**

*Note:* Boldface indicates statistical significance (*p*<0.001).

HR, hazard ratio; PC, principal component.

a Reported results are based on the first imputed data set.

b All Cox proportional-hazards regressions were adjusted for the 20 first PCs, third-degree relatedness, age, sex, education, and marital status.

c Model 2 included adjustments of Model 1, polygenic risk, and the number of alleles used to compute the polygenic risk score.

d Model 3 included adjustments of Model 2 and individual-level socioeconomic deprivation.

Dementia risk also increased monotonically across individual-level deprivation categories. Of participants with high individual-level deprivation, 1.41% developed dementia (95% CI=1.29, 1.53) (Appendix Table 3, available online) versus 0.44% (95% CI=0.38, 0.51) with low individual-level deprivation (adjusted HR=2.57, 95% CI=2.14, 3.08) (Table 3). Inclusion of genetic risk resulted in an adjusted HR of 2.57 (95% CI=2.14, 3.09) for high individual-level deprivation, indicating that individual-level deprivation is independent of genetic risk. Additional inclusion of area-level deprivation resulted in an adjusted HR of 2.38 (95% CI=1.98, 2.87) for high individual-level deprivation, suggesting that the association between individual-level deprivation and dementia risk is independent of area-level deprivation.

**Table 3:** Risk of Incident Dementia According to Individual-Level Socioeconomic Deprivation

Individual-level socioeconomic deprivation^a^	Model 1^b^	Model 1^b^	Model 1^b^	Model 2^c^	Model 2^c^	Model 2^c^	Model 3^d^	Model 3^d^	Model 3^d^
	Low (*n*=39,274)	Intermediate (*n*=117,821)	High (*n*=39,273)	Low (*n*=39,274)	Intermediate (*n*=117,821)	High (*n*=39,273)	Low (*n*=39,274)	Intermediate (*n*=117,821)	High (*n*=39,273)
Number of dementia cases/person-years^a^	174/309,221	1,042/929,551	553/306,541	174/309,221	1,042/929,551	553/306,541	174/309,221	1,042/929,551	553/306,541
HR (95% CI)	1 (ref)	1.63 (1.38, 1.93)	2.57 (2.14, 3.08)	1 (ref)	1.63 (1.38, 1.93)	2.57 (2.14, 3.09)	1 (ref)	1.62 (1.37, 1.92)	2.38 (1.98, 2.87)
*p*-value		**<0.001**	**<0.001**		**<0.001**	**<0.001**		**<0.001**	**<0.001**
*p*-value of trend^e^			**<0.001**			**<0.001**			**<0.001**

*Note:* Boldface indicates statistical significance (*p*<0.001).

HR, hazard ratio; PC, principal component.

a Reported results are based on the first imputed data set.

b All Cox proportional-hazards regressions were adjusted for the 20 first PCs, third-degree relatedness, age, sex, education, and marital status.

c Model 2 included adjustments of Model 1, polygenic risk, and the number of alleles used to compute the polygenic risk score.

d Model 3 included adjustments of Model 2 and area-level socioeconomic deprivation.

e *p*-value for trend was assessed using the continuous score of individual-level socioeconomic deprivation.

**Figure 1:**
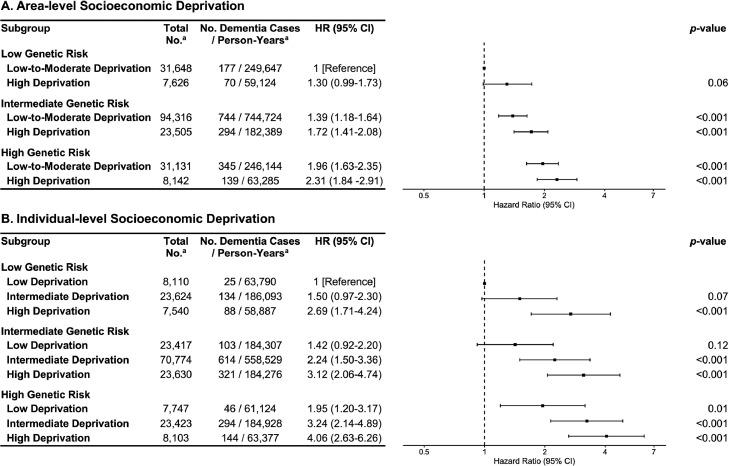
Risk of incident dementia for (A) area-level and (B) individual-level socioeconomic deprivation with genetic risk. *Note.* HRs correspond to combined groups of socioeconomic deprivation and genetic risk. All Cox proportional-hazards regressions were adjusted for the 20 first PCs, third-degree relatedness, number of alleles used to compute PRS, age, sex, education, marital status, healthy lifestyle, and depressive symptoms in the last 2 weeks. In addition, adjustments for (A) individual-level and (B) area-level socioeconomic deprivation were included. The number of dementia cases and dementia cases per person-years are based on the first imputed data set. ^a^Reported results are based on the first imputed data set. HR, hazard ratio; PC, principal component; PRS, polygenic risk score.

In models adjusted for socioeconomic deprivation, intermediate (adjusted HR=1.37, 95% CI=1.19, 1.58) and high (adjusted HR=1.91, 95% CI=1.63, 2.23) genetic risk were significantly associated with dementia risk. When genetic risk and deprivation categories were combined, there was a consistent pattern of increasing dementia risk (Figure 1). Of participants with high genetic risk and high area-level deprivation, 1.71% (95% CI=1.44, 2.01) (Appendix Table 4, available online) developed dementia versus 0.56% (95% CI=0.48, 0.65) with low genetic risk and low-to-moderate area-level deprivation (adjusted HR=2.31, 95% CI=1.84, 2.91). There was no significant interaction between area-level deprivation and genetic risk (*p*=0.77) (Appendix Figure 3, available online), indicating that the association with area-level deprivation did not vary substantially on the basis of genetic risk. Of participants with high genetic risk and high individual-level deprivation, 1.78% (95% CI=1.50, 2.09) (Appendix Table 5, available online) developed dementia versus 0.31% (95% CI=0.20, 0.45) with low genetic risk and low individual-level deprivation (adjusted HR=4.06, 95% CI=2.63, 6.26). There was no significant interaction between individual-level deprivation and genetic risk (*p*=0.07) (Appendix Figure 4, available online), indicating that the association with individual-level deprivation did not vary substantially on the basis of genetic risk.

The imaging substudy comprised 11,083 participants (mean [SD] age at imaging assessment=72.0 [3.2] years; 46.4% female) with available neuroimaging data. Total burden of WMH was higher in participants with high than in those with low-to-moderate, area-level deprivation (standardized coefficient=0.08, 95% CI=0.01, 0.15). Total burden of WMH was also higher in participants with high (standardized coefficient=0.10, 95% CI=0.01, 0.19) or intermediate (standardized coefficient=0.05, 95% CI=0.00, 0.10) than in those with low, individual-level deprivation. In participants with high area-level deprivation, gray matter volume was lower (standardized coefficient= –0.11, 95% CI= –0.18, –0.04). There were no significant associations with hippocampal, white matter, or whole brain volumes (Appendix Tables 6–11, available online).

In complete-case data, participants with high genetic risk and high individual-level or area-level deprivation were at higher risk of dementia (Appendix Tables 12–13, available online). Analysis in subsamples stratified by polygenic risk indicated that participants with high area-level or intermediate individual-level deprivation had a higher risk of dementia in intermediate and high but not in low genetic risk (Appendix Tables 14–15, available online). Participants with high individual-level deprivation had a higher risk of dementia in all genetic risk groups. Analysis in subsamples stratified by sex yielded a similar pattern of results (Appendix Tables 16–17, available online). Stroke may be on the causal pathway between deprivation and dementia and was therefore not included in the analyses; however, including a history of stroke led to practically identical result patterns.

For the imaging substudy, result patterns were replicated, using a less conservative set of potential imaging-related confounders, except for a nonsignificant association of intermediate individual-level deprivation with WMH and a significant association with gray matter volume. In complete-case data, the association of high individual-level and high area-level deprivation with WMH burden and the association of high area-level deprivation with gray matter volume were not significant (Appendix Tables 6–11, available online).

## DISCUSSION

Individual-level and area-level socioeconomic deprivation were associated with the risk of incident all-cause dementia, regardless of genetic risk. Participants with high genetic risk and area-level deprivation had a significantly higher risk of incident dementia than those with low genetic risk and low-to-moderate area-level deprivation, respectively. Similarly, participants with high genetic risk and individual-level deprivation had a significantly higher risk of incident dementia than those with low genetic risk and individual-level deprivation.

Previous studies had established that both area-level and individual-level deprivation were associated with an increased risk of dementia.^6^, ^7^, ^8^ Likewise, a previous meta-analysis of genome-wide association studies had established that a large proportion of the risk of developing late-onset AD is genetically determined.^2^ Risk was highest in high socioeconomic deprivation and genetic risk. This finding is in line with that of a previous study, which however additionally found a significant interaction of lower wealth with polygenic risk of dementia accelerating the time to diagnosis, possibly owing to differences in genetic risk assessment and strategy of data analysis.^6^^,^^46^ This study therefore extends previous findings by confirming pre-established associations and establishing that socioeconomic deprivation does not interact with genetic risk. In comparison, this study is considerably larger, incorporates a more comprehensive measure of genetic risk and potential mediators, and tests moderation of socioeconomic deprivation more comprehensively.

Individual-level deprivation was more robustly associated with dementia risk than area-level deprivation. Components of individual-level deprivation such as low income may increase dementia risk through reduced access to health care, poor-quality nutrition, and reduced cognitive stimulation that cannot be as effectively accounted for by area-level measures. Although there was a monotonic trend for individual-level deprivation, no such trend was found for area-level deprivation. This is in line with previous findings suggesting detrimental associations of neighborhood socioeconomic deprivation with health outcomes at the highest levels.^16^

Contrary to earlier findings, area-level associations remained associated with increased dementia risk after adjusting for individual-level deprivation.^7^ Area-level deprivation may capture dementia risk factors that are not fully explained by individual-level deprivation. Indeed, recent research suggests potential causal pathways through cognitive stimulation at large, access to residential green space, or air pollution.^11^^,^^13^^,^^14^ As such, area-level deprivation may reflect environments with limited opportunities for cognitive stimulation, healthy nutrition, or physical exercise.

The imaging substudy explored measures of brain health that might underlie increased dementia risk associated with socioeconomic deprivation. Higher area-level and individual-level deprivation were associated with greater WMH burden. WMHs are a well-established indicator of cerebral small vessel disease, double the risk of dementia, and are associated with more aggressive cognitive decline in patients with mild cognitive impairment.^17^^,^^47^ These results suggest a vascular pathway to dementia that might include both individual-level vascular risk factors, such as blood pressure, and area-level risk factors such as air pollution. Importantly, these risk factors are modifiable.^1^ High area-level deprivation was further associated with lower gray matter volume, suggesting additional, potentially neurodegenerative pathways. Although the results for hippocampal volume are inconclusive, lacking associations with other markers of brain health typically associated with dementia risk suggests global effects of area-level deprivation that might represent accelerated brain aging.

### Limitations

Some limitations should be considered. First, individual-level and area-level deprivation are correlated (*r*=0.33). Second, residual confounding may exist despite careful confounder adjustment. Third, reverse causation cannot be ruled out, despite a median follow-up of 8 years. Fourth, 1 in 71 participants aged >65 years was ascertained with dementia compared with 1 in 14 in the general population, suggesting a healthy volunteer bias.^48^ Representativeness is further limited owing to a low response rate to the invitation to participate in the UK Biobank study. Nonetheless, previous findings suggest that health hazards correspond to findings in representative samples.^23^^,^^49^^,^^50^ Fifth, without case finding, sensitivity cannot be tested, and dementia may have not been detected in all cases. In addition, ascertainment in hospital and death records may select more severe cases, potentially biasing estimates.^22^ Sixth, estimates may be biased because the competing risk of death can precede dementia diagnosis.^51^^,^^52^ Seventh, analyses were restricted to those aged ≥60 years, limiting cases, and to European ancestry, limiting generalizability. Finally, some associations were not replicated in complete-case data, likely owing to disproportionally missing data in higher deprivation (Appendix Table 18, available online).

## CONCLUSIONS

In older adults without dementia, area-level and individual-level socioeconomic deprivation and genetic risk were significantly and independently associated with a higher risk of dementia. Dementia prevention interventions may be particularly effective if targeted to people living in households and areas with fewer socioeconomic resources, regardless of genetic vulnerability.

## Appendix SUPPLEMENTAL MATERIAL

Image, application 1

## Footnotes

Supplemental materials associated with this article can be found in the online version at https://doi.org/10.1016/j.amepre.2023.01.012.
