# Supplementary material for: Socioeconomic Deprivation, Genetic Risk, and Incident Dementia
Source: Am J Prev Med. 2023 May;64(5):621–30. doi: 10.1016/j.amepre.2023.01.012 (PMC10126314; doi:10.1016/j.amepre.2023.01.012)
Supplement: Supplementary file 1 [file mmc1.pdf]

Appendix

Klee, M, Leist, A. K., Veldsman, M., Ranson, J. M., Llewellyn, D. J. (Manuscript submitted for publication). Socioeconomic Deprivation, Genetic Risk, and Incident Dementia. *American Journal of Preventive Medicine.*

Appendix 1. Tables

Appendix Table 1. Coefficients of Cox Proportional-Hazards Regressions Used for Weighting of the Individual-Level Socioeconomic Deprivation Score

Appendix Table 2. Total Participants and Incident Dementia Cases in Area-Level Socioeconomic Deprivation Groups

Appendix Table 3. Total Participants and Incident Dementia Cases in Individual-Level Socioeconomic Deprivation Groups

Appendix Table 4. Total Participants and Incident Dementia Cases According to Area-Level Socioeconomic Deprivation within Each Genetic Risk Category

Appendix Table 5. Total Participants and Incident Dementia Cases According to Individual-Level Socioeconomic Deprivation within Each Genetic Risk Category

Appendix Table 6. Coefficients for Multivariable Linear Regressions of White Matter Hyperintensities in Imputed and Complete-Case Data with Full and Reduced Deconfounding Set

Appendix Table 7. Coefficients for Multivariable Linear Regressions of Hippocampal Volume (r) in Imputed and Complete-Case Data with Full and Reduced Deconfounding Set

Appendix Table 8. Coefficients for Multivariable Linear Regressions of Hippocampal Volume (l) in Imputed and Complete-Case Data with Full and Reduced Deconfounding Set

Appendix Table 9. Coefficients for Multivariable Linear Regressions of Whole Brain Volume in Imputed and Complete-Case Data with Full and Reduced Deconfounding Set

Appendix Table 10. Coefficients for Multivariable Linear Regressions of White Matter Volume in Imputed and Complete-Case Data with Full and Reduced Deconfounding Set

Appendix Table 11. Coefficients for Multivariable Linear Regressions of Grey Matter Volume in Imputed and Complete-Case Data with Full and Reduced Deconfounding Set

Appendix Table 12. Risk of Incident Dementia According to Combined Area-Level Socioeconomic Deprivation and Genetic Risk in Complete-Case Data

Appendix Table 13. Risk of Incident Dementia According to Combined Individual-Level Socioeconomic Deprivation and Genetic Risk in Complete-Case Data

Appendix Table 14. Risk of Dementia According to Area-Level Socioeconomic Deprivation in Subgroups Stratified by Genetic Risk

Appendix Table 15. Risk of Dementia According to Individual-Level Socioeconomic Deprivation in Subgroups Stratified by Genetic Risk

Appendix Table 16. Risk of Dementia According to Area-Level Socioeconomic Deprivation in Subgroups Stratified by Sex

Appendix Table 17. Risk of Dementia According to Individual-Level Socioeconomic Deprivation in Subgroups Stratified by Sex

Appendix Table 18. Proportion of Individual-Level Socioeconomic Deprivation Across Lifestyle Categories in Complete-Case Data and Imputed Data for the Full Sample and Imaging Subsample

Appendix 2. Figures

Appendix Figure 1. Risk of Incident Dementia by Area-Level Socioeconomic Deprivation Quintile

Appendix Figure 2. Proportion of Missing Data Prior to Imputation.

Appendix Figure 3. Risk of Incident Dementia by Area-Level Socioeconomic Deprivation and Genetic Risk Including Interaction Terms

Appendix Figure 4. Risk of Incident Dementia by Individual-Level Socioeconomic Deprivation and Genetic Risk Including Interaction Terms

Appendix 1. Tables

Appendix Table 1. Coefficients of Cox Proportional-Hazards Regressions Used for Weighting of the Individual-Level Socioeconomic Deprivation Score[a]

	Including not disclosed[b]			Excluding not disclosed[c]		
**Characteristic**	**Total No.[d]**	**Coefficient**	**P value**	**Total No.[d]**	**Coefficient**	**P value**
**Income[e]**						
**Greater 31,000**	53,346	0 [Reference]		53,146	0 [Reference]	
**From 18,000 to 31,000**	52,834	0.34 (0.18-0.50)	**<.001**	52,572	0.38 (0.21-0.54)	**<.001**
**Smaller 18,000**	54,866	0.48 (0.31-0.65)	**<.001**	54,339	0.54 (0.36-0.71)	**<.001**
**Not disclosed**	35,322	0.67 (0.50-0.85)	**<.001**			
**Housing Type**						
**House or Flat**	194,542	0 [Reference]		159,059	0 [Reference]	
**Other**	1,826	0.45 (0.11-0.78)	.01	998	0.74 (0.33-1.14)	**<.001**
**Home Ownership**						
**Own Outright**	151,971	0 [Reference]		123,790	0 [Reference]	
**Other**	44,397	0.35 (0.24-0.46)	**<.001**	36,267	0.39 (0.26-0.52)	**<.001**
**Car Ownership**						
**One or more**	179,091	0 [Reference]		146,778	0 [Reference]	
**Other**	17,277	0.32 (0.18-0.46)	**<.001**	13,279	0.29 (0.12-0.45)	**<.001**

Boldface indicates statistical significance (p<.001).

[a]All Cox proportional-hazards regressions were adjusted for the 20 first PCs, 3rd degree relatedness, age, sex, education, retirement status and number of people in the household.

[b]Coefficients used to compute individual-level socioeconomic deprivation with not disclosed information included in other category for housing type, home ownership and car ownership.

[c]Coefficients used to compute individual-level socioeconomic deprivation when excluding not disclosed information relating to income, housing type, home ownership and car ownership.

[d]Reported results are based on the first imputed data set.

[e]Income assessed in Pound sterling (£) based on average total household income before tax.

Appendix Table 2. Total Participants and Incident Dementia Cases in Area-Level Socioeconomic Deprivation Groups

Area-Level Socioeconomic Deprivation	Low-to- Moderate	High
No. of Dementia Cases[a]	1,266	503
Absolute Risk, % (95% CI)[a]	0.81 (0.76-0.85)	1.28 (1.17-1.40)
Incidence Rates per 1,000 Person-Years (95% CI)[a]	1.02 (0.97-1.08)	1.65 (1.51-1.80)
Total No.[a]	157,095	39,273

[a]Reported results are based on the first imputed data set.

Appendix Table 3. Total Participants and Incident Dementia Cases in Individual-Level Socioeconomic Deprivation Groups

Area-Level Socioeconomic Deprivation	Low	Intermediate	High
**No. of Dementia Cases[a]**	174	1,042	553
**Absolute Risk, % (95% CI)[a]**	0.44 (0.38-0.51)	0.88 (0.83-0.94)	1.41 (1.29-1.53)
**Incidence Rates per 1,000 Person-Years (95% CI)[a]**	0.56 (0.48-0.65)	1.12 (1.05-1.19)	1.80 (1.66-1.96)
**Total No.[a]**	39,274	117,821	39,273

[a]Reported results are based on the first imputed data set.

Appendix Table 4. Total Participants and Incident Dementia Cases According to Area-Level Socioeconomic Deprivation within Each Genetic Risk Category

Genetic risk	Low		Intermediate		High	
Area-Level Socioeconomic Deprivation	Low-to- Moderate	High	Low-to- Moderate	High	Low-to- Moderate	High
No. of Dementia Cases[a]	177	70	744	294	345	139
Absolute Risk, % (95% CI)[a]	0.56 (0.48-0.65)	0.92 (0.72-1.16)	0.79 (0.73-0.85)	1.25 (1.11-1.40)	1.11 (0.99-1.23)	1.71 (1.44-2.01)
Incidence Rates per 1,000 Person-Years (95% CI)[a]	0.71 (0.61-0.82)	1.18 (0.92-1.50)	1.00 (0.93-1.07)	1.61 (1.43-1.81)	1.40 (1.26-1.56)	2.20 (1.85-2.59)
Total No.[a]	31,648	7,626	94,316	23,505	31,131	8,142

[a]Reported results are based on the first imputed data set.

Appendix Table 5. Total Participants and Incident Dementia Cases According to Individual-Level Socioeconomic Deprivation within Each Genetic Risk Category

Genetic risk	Low			Intermediate			High		
**Individual-Level Socioeconomic Deprivation**	**Low**	**Intermediate**	**High**	**Low**	**Intermediate**	**High**	**Low**	**Intermediate**	**High**
**No. of Dementia Cases[a]**	25	134	88	103	614	321	46	294	144
**Absolute Risk, % (95% CI)[a]**	0.31 (0.20-0.45)	0.57 (0.48-0.67)	1.17 (0.94-1.44)	0.44 (0.36-0.53)	0.87 (0.80-0.94)	1.36 (1.21-1.51)	0.59 (0.44-0.79)	1.26 (1.12-1.41)	1.78 (1.50-2.09)
**Incidence Rates per 1,000 Person-Years (95% CI)[a]**	0.39 (0.25-0.58)	0.72 (0.60-0.85)	1.49 (1.20-1.84)	0.56 (0.46-0.68)	1.10 (1.01-1.19)	1.74 (1.56-1.94)	0.75 (0.55-1.00)	1.59 (1.41-1.78)	2.27 (1.92-2.67)
**Total No.[a]**	8,110	23,624	7,540	23,417	70,774	23,630	7,747	23,423	8,103

[a]Reported results are based on the first imputed data set.

Appendix Table 6. Coefficients for Multivariable Linear Regressions of White Matter Hyperintensities in Imputed and Complete-Case Data with Full and Reduced Deconfounding Set[a]

	Imputed Data				Complete-Case Data			
	Full Set		Reduced Set		Full Set		Reduced Set	
**Characteristic**	**Coefficient (95% CI)**	**P value**	**Coefficient (95% CI)**	**P value**	**Coefficient (95% CI)**	**P value**	**Coefficient (95% CI)**	**P value**
**Individual-Level Socioeconomic Deprivation**								
**Low**	0 [Reference]		0 [Reference]		0 [Referenc		0 [Referenc	
**Intermediate**	0.05 (0.00-0.10)	**.04**	0.04 (-0.01-0.09)	.09	0.06 (0.00-0.12)	**.04**	0.05 (-0.01-0.10)	.08
**High**	0.10 (0.01-0.19)	**.03**	0.10 (0.01-0.19)	**.03**	0.06 (-0.06-0.17)	.33	0.06 (-0.06-0.17)	.34
**Area-Level Socioeconomic Deprivation**								
**Low-to-Moderate**	0 [Reference]		0 [Reference]		0 [Reference]		0 [Reference]	
**High**	0.08 (0.01-0.15)	**.03**	0.07 (0.01-0.14)	**.03**	0.07 (-0.03-0.17)	.16	0.07 (-0.03-0.16)	.17
**Total No.**	11,035				8,131			

Abbreviation: CI, confidence interval. Boldface indicates statistical significance (p<.05).

[a]All imaging derived phenotypes were deconfounded in multivariable linear regressions, either adjusting for the full set including site-specific derivatives capturing indicators of age, age squared, sex, age-sex interactions, head size, days since the scanner start-up, days since the scanner start-up squared and two dummy variables coding site or the reduced set including age, sex, age-sex interactions, head size and two dummy variables coding site. Residuals were then entered in secondary multivariable linear regressions including 20 first PCs, 3rd degree relatedness, number of alleles used to compute the polygenic risk score, education, marital status, healthy lifestyle, depressive symptoms in last two weeks, individual-level and area-level socioeconomic deprivation as well as genetic risk.

Appendix Table 7. Coefficients for Multivariable Linear Regressions of Hippocampal Volume (r) in Imputed and Complete-Case Data with Full and Reduced Deconfounding Set[a]

	Imputed Data				Complete-Case Data			
	Full Set		Reduced Set		Full Set		Reduced Set	
**Characteristic**	**Coefficient (95% CI)**	**P value**	**Coefficient (95% CI)**	**P value**	**Coefficient (95% CI)**	**P value**	**Coefficient (95% CI)**	**P value**
**Individual-Level Socioeconomic Deprivation**								
**Low**	0 [Reference]		0 [Reference]		0 [Reference]		0 [Reference]	
**Intermediate**	-0.03 (-0.09-0.02)	.18	-0.04 (-0.09-0.01)	.15	-0.04 (-0.09-0.02)	.20	-0.04 (-0.10-0.02)	.16
**High**	-0.00 (-0.09-0.09)	.94	-0.01 (-0.10-0.08)	.88	0.04 (-0.07-0.15)	.50	0.03 (-0.08-0.15)	.55
**Area-Level Socioeconomic Deprivation**								
**Low-to-Moderate**	0 [Reference]		0 [Reference]		0 [Reference]		0 [Reference]	
**High**	-0.04 (-0.11-0.03)	.26	-0.04 (-0.11-0.03)	.27	0.00 (-0.09-0.09)	.98	-0.00 (-0.09-0.09)	1.00
**Total No.**	10,838				7,999			

Abbreviation: CI, confidence interval. Boldface indicates statistical significance (p<.05).

[a]All imaging derived phenotypes were deconfounded in multivariable linear regressions, either adjusting for the full set including site-specific derivatives capturing indicators of age, age squared, sex, age-sex interactions, head size, days since the scanner start-up, days since the scanner start-up squared and two dummy variables coding site or the reduced set including age, sex, age-sex interactions, head size and two dummy variables coding site. Residuals were then entered in secondary multivariable linear regressions including 20 first PCs, 3rd degree relatedness, number of alleles used to compute the polygenic risk score, education, marital status, healthy lifestyle, depressive symptoms in last two weeks, individual-level and area-level socioeconomic deprivation as well as genetic risk.

Appendix Table 8. Coefficients for Multivariable Linear Regressions of Hippocampal Volume (l) in Imputed and Complete-Case Data with Full and Reduced Deconfounding Set[a]

	Imputed Data				Complete-Case Data			
	Full Set		Reduced Set		Full Set		Reduced Set	
**Characteristic**	**Coefficient (95% CI)**	**P value**	**Coefficient (95% CI)**	**P value**	**Coefficient (95% CI)**	**P value**	**Coefficient (95% CI)**	**P value**
**Individual-Level Socioeconomic Deprivation**								
**Low**	0 [Reference]		0 [Reference]		0 [Reference]		0 [Reference]	
**Intermediate**	-0.05 (-0.10-0.00)	.06	-0.05 (-0.10-0.00)	.06	-0.03 (-0.08-0.03)	.37	-0.03 (-0.08-0.03)	.32
**High**	-0.01 (-0.09-0.08)	.91	-0.01 (-0.10-0.09)	.91	0.07 (-0.04-0.18)	.22	0.07 (-0.04-0.18)	.22
**Area-Level Socioeconomic Deprivation**								
**Low-to-Moderate**	0 [Reference]		0 [Reference]		0 [Reference]		0 [Reference]	
**High**	-0.06 (-0.13-0.01)	.07	-0.06 (-0.13-0.01)	.08	-0.05 (-0.14-0.04)	.26	-0.05 (-0.14-0.04)	.29
**Total No.**	10,920				8,056			

Abbreviation: CI, confidence interval. Boldface indicates statistical significance (p<.05).

[a]All imaging derived phenotypes were deconfounded in multivariable linear regressions, either adjusting for the full set including site-specific derivatives capturing indicators of age, age squared, sex, age-sex interactions, head size, days since the scanner start-up, days since the scanner start-up squared and two dummy variables coding site or the reduced set including age, sex, age-sex interactions, head size and two dummy variables coding site. Residuals were then entered in secondary multivariable linear regressions including 20 first PCs, 3rd degree relatedness, number of alleles used to compute the polygenic risk score, education, marital status, healthy lifestyle, depressive symptoms in last two weeks, individual-level and area-level socioeconomic deprivation as well as genetic risk.

Appendix Table 9. Coefficients for Multivariable Linear Regressions of Whole Brain Volume in Imputed and Complete-Case Data with Full and Reduced Deconfounding Set[a]

	Imputed Data				Complete-Case Data			
	Full Set		Reduced Set		Full Set		Reduced Set	
**Characteristic**	**Coefficient (95% CI)**	**P value**	**Coefficient (95% CI)**	**P value**	**Coefficient (95% CI)**	**P value**	**Coefficient (95% CI)**	**P value**
**Individual-Level Socioeconomic Deprivation**								
**Low**	0 [Reference]		0 [Reference]		0 [Reference]		0 [Reference]	
**Intermediate**	-0.03 (-0.08-0.02)	.25	-0.03 (-0.08-0.02)	.30	-0.03 (-0.08-0.03)	.31	-0.02 (-0.08-0.03)	.39
**High**	-0.03 (-0.12-0.06)	.46	-0.03 (-0.12-0.06)	.49	0.07 (-0.03-0.18)	.17	0.08 (-0.03-0.18)	.15
**Area-Level Socioeconomic Deprivation**								
**Low-to-Moderate**	0 [Reference]		0 [Reference]		0 [Reference]		0 [Reference]	
**High**	-0.05 (-0.12-0.02)	.17	-0.05 (-0.12-0.02)	.19	0.00 (-0.09-0.09)	.99	-0.00 (-0.09-0.09)	.94
**Total No.**	11,035				8,139			

Abbreviation: CI, confidence interval. Boldface indicates statistical significance (p<.05).

[a]All imaging derived phenotypes were deconfounded in multivariable linear regressions, either adjusting for the full set including site-specific derivatives capturing indicators of age, age squared, sex, age-sex interactions, head size, days since the scanner start-up, days since the scanner start-up squared and two dummy variables coding site or the reduced set including age, sex, age-sex interactions, head size and two dummy variables coding site. Residuals were then entered in secondary multivariable linear regressions including 20 first PCs, 3rd degree relatedness, number of alleles used to compute the polygenic risk score, education, marital status, healthy lifestyle, depressive symptoms in last two weeks, individual-level and area-level socioeconomic deprivation as well as genetic risk.

Appendix Table 10. Coefficients for Multivariable Linear Regressions of White Matter Volume in Imputed and Complete-Case Data with Full and Reduced Deconfounding Set[a]

	Imputed Data				Complete-Case Data			
	Full Set		Reduced Set		Full Set		Reduced Set	
**Characteristic**	**Coefficient (95% CI)**	**P value**	**Coefficient (95% CI)**	**P value**	**Coefficient (95% CI)**	**P value**	**Coefficient (95% CI)**	**P value**
**Individual-Level Socioeconomic Deprivation**								
**Low**	0 [Reference]		0 [Reference]		0 [Reference]		0 [Reference]	
**Intermediate**	-0.00 (-0.05-0.05)	.91	0.01 (-0.04-0.06)	.82	0.01 (-0.04-0.06)	.75	0.02 (-0.04-0.07)	.50
**High**	-0.02 (-0.10-0.06)	.60	-0.02 (-0.10-0.07)	.67	0.06 (-0.05-0.16)	.30	0.06 (-0.04-0.17)	.26
**Area-Level Socioeconomic Deprivation**								
**Low-to-Moderate**	0 [Reference]		0 [Reference]		0 [Reference]		0 [Reference]	
**High**	0.02 (-0.05-0.09)	.63	0.02 (-0.05-0.09)	.59	0.02 (-0.07-0.12)	.65	0.02 (-0.08-0.11)	.74
**Total No.**	11,039				8,140			

Abbreviation: CI, confidence interval. Boldface indicates statistical significance ($p<.05$).

[a]All imaging derived phenotypes were deconfounded in multivariable linear regressions, either adjusting for the full set including site-specific derivatives capturing indicators of age, age squared, sex, age-sex interactions, head size, days since the scanner start-up, days since the scanner start-up squared and two dummy variables coding site or the reduced set including age, sex, age-sex interactions, head size and two dummy variables coding site. Residuals were then entered in secondary multivariable linear regressions including 20 first PCs, 3rd degree relatedness, number of alleles used to compute the polygenic risk score, education, marital status, healthy lifestyle, depressive symptoms in last two weeks, individual-level and area-level socioeconomic deprivation as well as genetic risk.

Appendix Table 11. Coefficients for Multivariable Linear Regressions of Grey Matter Volume in Imputed and Complete-Case Data with Full and Reduced Deconfounding Set[a]

	Imputed Data				Complete-Case Data			
	Full Set		Reduced Set		Full Set		Reduced Set	
**Characteristic**	**Coefficient (95% CI)**	**P value**	**Coefficient (95% CI)**	**P value**	**Coefficient (95% CI)**	**P value**	**Coefficient (95% CI)**	**P value**
**Individual-Level Socioeconomic Deprivation**								
**Low**	0 [Reference]		0 [Reference]		0 [Reference]		0 [Reference]	
**Intermediate**	-0.05 (-0.10-0.00)	.07	-0.05 (-0.10-0.00)	**.04**	-0.06 (-0.12--0.00)	**.046**	-0.06 (-0.12--0.01)	**.03**
**High**	-0.04 (-0.14-0.05)	.39	-0.04 (-0.14-0.05)	.35	0.04 (-0.07-0.14)	.50	0.03 (-0.07-0.14)	.55
**Area-Level Socioeconomic Deprivation**								
**Low-to-Moderate**	0 [Reference]		0 [Reference]		0 [Reference]		0 [Reference]	
**High**	-0.11 (-0.18–0.04)	**.004**	-0.11 (-0.18–0.03)	**.004**	-0.04 (-0.13-0.06)	.43	-0.04 (-0.13-0.06)	.43
**Total No.**	11,018				8,128			

Abbreviation: CI, confidence interval. Boldface indicates statistical significance ($p<.05$).

[a]All imaging derived phenotypes were deconfounded in multivariable linear regressions, either adjusting for the full set including site-specific derivatives capturing indicators of age, age squared, sex, age-sex interactions, head size, days since the scanner start-up, days since the scanner start-up squared and two dummy variables coding site or the reduced set including age, sex, age-sex interactions, head size and two dummy variables coding site. Residuals were then entered in secondary multivariable linear regressions including 20 first PCs, 3rd degree relatedness, number of alleles used to compute the polygenic risk score, education, marital status, healthy lifestyle, depressive symptoms in last two weeks, individual-level and area-level socioeconomic deprivation as well as genetic risk.

Appendix Table 12. Risk of Incident Dementia According to Combined Area-Level Socioeconomic Deprivation and Genetic Risk in Complete-Case Data[a]

**Genetic risk**	**Low**		**Intermediate**		**High**	
**Area-Level Socioeconomic Deprivation**	**Low-to-Moderate**	**High**	**Low-to-Moderate**	**High**	**Low-to-Moderate**	**High**
**Total No.**	22,154	3,875	66,516	11,927	21,778	4,055
**No. of Dementia Cases / Person-Years**	120 / 173,636	31 / 29,809	488 / 521,545	138 / 92,019	215 / 171,055	50 / 31,449
**HR (95% CI)**	1 [Reference]	1.26 (0.85-1.88)	1.34 (1.10-1.64)	1.76 (1.37-2.25)	1.81 (1.45-2.27)	1.80 (1.29-2.52)
**P Value**		.25	**.004**	**<.001**	**<.001**	**<.001**

Abbreviation: HR, hazard ratio. Boldface indicates statistical significance (p<.05).

[a]Cox proportional-hazards regression model was adjusted for the 20 first PCs, 3rd degree relatedness, number of alleles used to compute the polygenic risk score, age, sex, education, marital status, healthy lifestyle, depressive symptoms in last two weeks and individual-level socioeconomic deprivation.

Appendix Table 13. Risk of Incident Dementia According to Combined Individual-Level Socioeconomic Deprivation and Genetic Risk in Complete-Case Data[a]

	Low			Intermediate			High		
**Individual-Level Socioeconomic Deprivation**	**Low**	**Intermediate**	**High**	**Low**	**Intermediate**	**High**	**Low**	**Intermediate**	**High**
**Total No.**	6,849	15,696	3,484	19,790	47,819	10,834	6,554	15,593	3,686
**No. of Dementia Cases / Person-Years**	23 / 53,656	89 / 122,655	39 / 27,133	84 / 155,026	399 / 374,404	143 / 84,132	39 / 51,460	174 / 122,193	52 / 28,852
**HR (95% CI)**	1 [Reference]	1.37 (0.86-2.17)	2.29 (1.36-3.86)	1.26 (0.79-2.00)	1.97 (1.29-3.01)	2.75 (1.75-4.31)	1.80 (1.07-3.01)	2.63 (1.69-4.08)	2.89 (1.75-4.76)
**P Value**		.18	**.002**	.33	**.002**	**<.001**	**.03**	**<.001**	**<.001**

Abbreviation: HR, hazard ratio. Boldface indicates statistical significance (p<.05).

[a]Cox proportional-hazards regression model was adjusted for the 20 first PCs, 3rd degree relatedness, number of alleles used to compute the polygenic risk score, age, sex, education, marital status, healthy lifestyle, depressive symptoms in last two weeks and area-level socioeconomic deprivation.

Appendix Table 14. Risk of Dementia According to Area-Level Socioeconomic Deprivation in Subgroups Stratified by Genetic Risk[a]

Genetic Risk	Low		Intermediate		High	
**Area-Level Socioeconomic Deprivation[b]**	**Low-to-Moderate (n = 31,648)**	**High (n = 7,626)**	**Low-to-Moderate (n = 94,316)**	**High (n = 23,505)**	**Low-to-Moderate (n = 31,131)**	**High (n = 8,142)**
**No. of Dementia Cases / Person-Years[b]**	177 / 249,647	70 / 59,124	744 / 744,724	294 / 182,389	345 / 246,144	139 / 63,285
**HR (95% CI)**	1 [Reference]	1.18 (0.87-1.61)	1 [Reference]	1.29 (1.11-1.50)	1 [Reference]	1.32 (1.06-1.64)
**P Value**		.29		**<.001**		**.01**

Abbreviation: HR, hazard ratio. Boldface indicates statistical significance (p<.05).

[a]All Cox proportional-hazards regressions were adjusted for the 20 first PCs, 3rd degree relatedness, number of alleles used to compute the polygenic risk score, age, sex, education, marital status and individual-level socioeconomic deprivation.

[b]Reported results are based on the first imputed data set.

Appendix Table 15. Risk of Dementia According to Individual-Level Socioeconomic Deprivation in Subgroups Stratified by Genetic Risk[a]

Genetic Risk	Low			Intermediate			High		
**Individual-Level Socioeconomic Deprivation[b]**	**Low (n = 8,110)**	**Intermediate (n = 23,624)**	**High (n = 7,540)**	**Low (n = 23,417)**	**Intermediate (n = 70,774)**	**High (n = 23,630)**	**Low (n = 7,747)**	**Intermediate (n = 23,423)**	**High (n = 8,103)**
**No. of Dementia Cases / Person-Years[b]**	25 / 63,790	134 / 186,093	88 / 58,887	103 / 184,307	614 / 558,529	321 / 184,276	46 / 61,124	294 / 184,928	144 / 63,377
**HR (95% CI)**	1 [Reference]	1.50 (0.96-2.35)	2.73 (1.66-4.50)	1 [Reference]	1.61 (1.29-2.00)	2.34 (1.83-2.99)	1 [Reference]	1.72 (1.25-2.38)	2.31 (1.61-3.32)
**P Value**		.07	**<.001**		**<.001**	**<.001**		**<.001**	**<.001**
**P Value for Trend**	**<.001**			**<.001**			**<.001**		

Abbreviation: HR, hazard ratio. Boldface indicates statistical significance (p<.05).

[a]All Cox proportional-hazards regressions were adjusted for the 20 first PCs, 3rd degree relatedness, number of alleles used to compute the polygenic risk score, age, sex, education, marital status and area-level socioeconomic deprivation.

[b]Reported results are based on the first imputed data set.

Appendix Table 16. Risk of Dementia According to Area-Level Socioeconomic Deprivation in Subgroups Stratified by Sex[a]

**Sex**	**Female**		**Male**	
**Area-Level Socioeconomic Deprivation[b]**	**Low-to-Moderate (n = 82,938)**	**High (n = 20,496)**	**Low-to-Moderate (n = 74,157)**	**High (n = 18,777)**
**No. of Dementia Cases / Person-Years[b]**	569 / 659,247	221 / 161,207	697 / 581,268	282 / 143,591
**HR (95% CI)**	1 [Reference]	1.25 (1.05-1.48)	1 [Reference]	1.31 (1.12-1.53)
**P Value**		**.01**		**<.001**

Abbreviation: HR, hazard ratio. Boldface indicates statistical significance (p<.05).

[a]All Cox proportional-hazards regressions were adjusted for the 20 first PCs, 3rd degree relatedness, number of alleles used to compute the polygenic risk score, genetic risk, age, sex, education, marital status and individual-level socioeconomic deprivation.

[b]Reported results are based on the first imputed data set.

Appendix Table 17. Risk of Dementia According to Individual-Level Socioeconomic Deprivation in Subgroups Stratified by Sex[a]

Sex	Female			Male		
**Individual-Level Socioeconomic Deprivation[b]**	**Low (n = 16,617)**	**Intermediate (n = 65,137)**	**High (n = 21,680)**	**Low (n = 22,657)**	**Intermediate (n = 52,684)**	**High (n = 17,593)**
**No. of Dementia Cases / Person- Years[b]**	57 / 131,217	467 / 517,550	266 / 171,687	117 / 178,004	575 / 412,001	287 / 134,854
**HR (95% CI)**	1 [Reference]	1.51 (1.14-2.00)	2.29 (1.68-3.13)	1 [Reference]	1.71 (1.39-2.10)	2.44 (1.92-3.11)
**P Value**		**.004**	**<.001**		**<.001**	**<.001**
**P Value for Trend**	**<.001**			**<.001**		

Abbreviation: HR, hazard ratio. Boldface indicates statistical significance (p<.05).

[a]All Cox proportional-hazards regressions were adjusted for the 20 first PCs, 3rd degree relatedness, number of alleles used to compute the polygenic risk score, genetic risk, age, sex, education, marital status and area-level socioeconomic deprivation.

[b]Reported results are based on the first imputed data set.

Appendix Table 18. Proportion of Individual-Level Socioeconomic Deprivation Across Lifestyle Categories in Complete-Case Data and Imputed Data for the Full Sample and Imaging Subsample

Complete-Case Data										
	Individual-Level Socioeconomic Deprivation – Full Sample					Individual-Level Socioeconomic Deprivation – Imaging Subsample				
Lifestyle[b]	Low	Intermediate	High	Missing No.	Total No.[c]	Low	Intermediate	High	Missing No.	Total No.[c]
Favourable	19.79%	60.13%	20.08%	79	32,761	29.27%	58.53%	12.20%	8	2,033
Intermediate	22.94%	61.06%	15.99%	419	98,104	33.68%	58.40%	7.92%	29	6,123
Unfavourable	20.94%	56.87%	22.19%	282	32,559	37.70%	54.00%	8.30%	8	1,626
Missing	10.21%	59.79%	30.00%	250	31,914	19.39%	65.46%	15.14%	8	1,248
Imputed Data[a]										
	Individual-Level Socioeconomic Deprivation – Full Sample					Individual-Level Socioeconomic Deprivation – Imaging Subsample				
Lifestyle[b]	Low	Intermediate	High		Total No.	Low	Intermediate	High		Total No.
Favourable	17.44%	64.87%	17.69%		39,273	26.52%	61.94%	11.54%		2,244
Intermediate	20.90%	60.26%	18.84%		117,821	32.53%	57.05%	10.42%		6,898
Unfavourable	19.88%	54.35%	25.78%		39,274	36.32%	52.24%	11.44%		1,941

[a]Reported results are based on the first imputed data set.

[b]Percentages are based on the total number of participants without missing data on individual-level deprivation and may not sum to 100 because of rounding.

[c]Total number of participants without missing data on individual-level deprivation.

Appendix 2. Figures

Appendix Figure 1. Risk of Incident Dementia According to Area-Level Socioeconomic Deprivation Quintiles

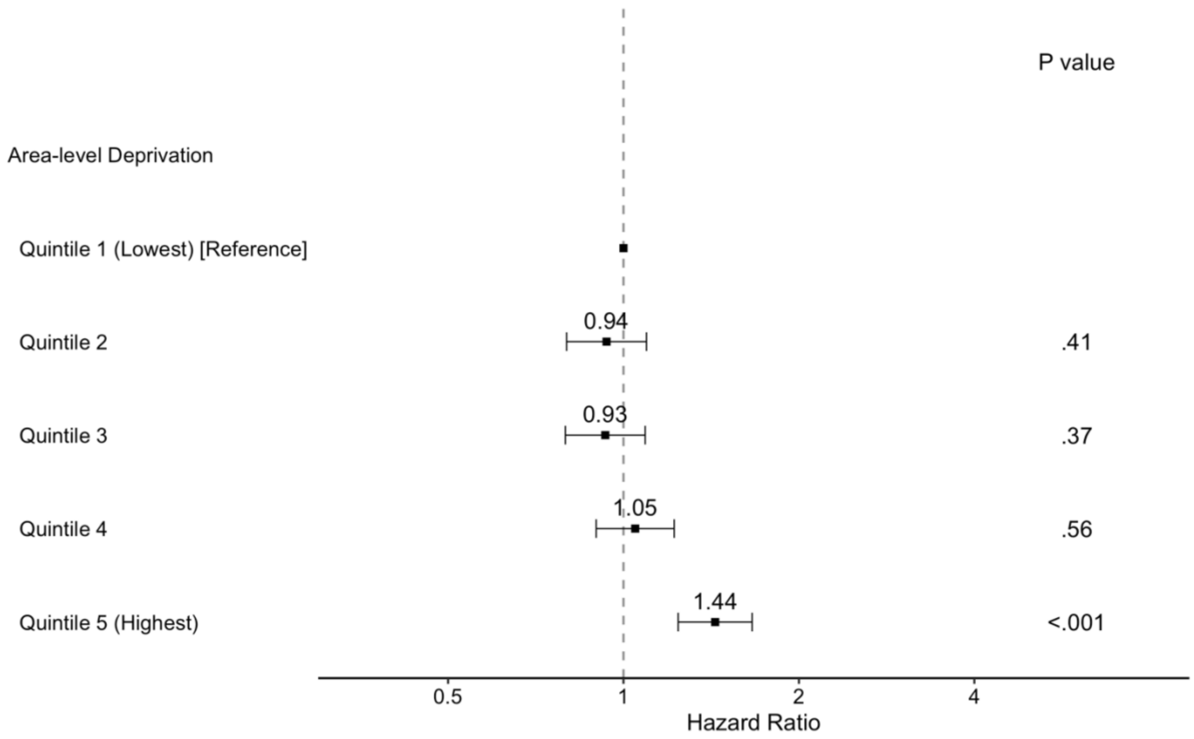

Note. Bars indicate 95% confidence intervals. Hazard ratios are depicted on a log-scale. All Cox proportional-hazards regressions were adjusted for the 20 first PCs, 3rd degree relatedness, age, sex, education and marital status.

Appendix Figure 2. Proportion of Missing Data Prior to Imputation.

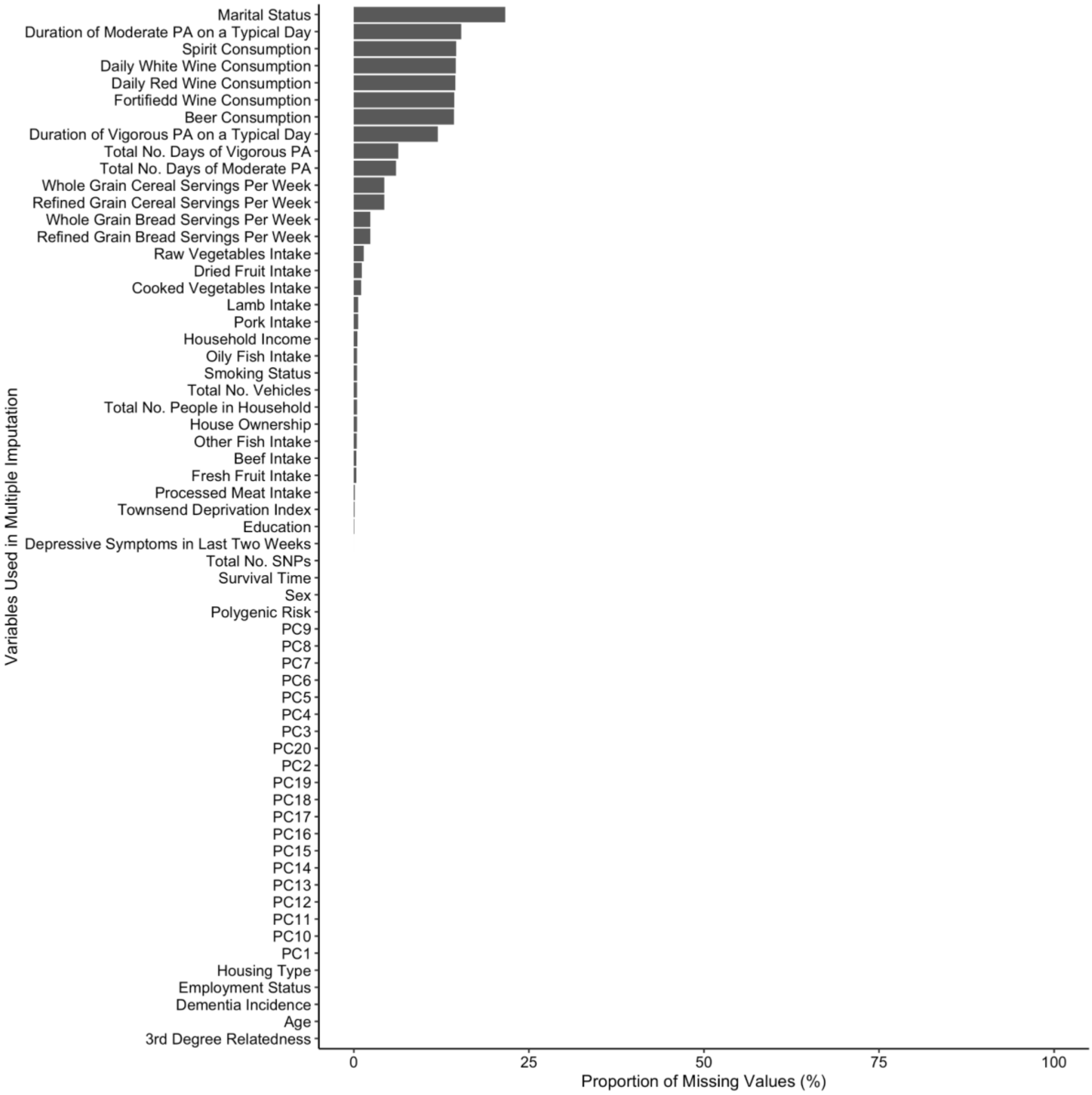

Note. All variable relevant to our analyses were used to impute missing values, including 29 variables were complete after application of eligibility criteria. Some variables were considered relevant to all analyses and thus used for imputation of all variables: 20 first PCs, 3rd degree relatedness, number of alleles used to compute the polygenic risk score, polygenic risk score, age, sex, education, dementia, follow-up time, household income, vehicle and home ownership, housing type, Townsend deprivation index, and number of people in the household. Further variables used for during imputation were retirement status, marital status, depressive symptoms in last two weeks and 24 variables indicating physical activity, diet, smoking behavior and alcohol intake, used to compute the healthy lifestyle index.

Appendix Figure 3. Risk of Incident Dementia by Area-Level Socioeconomic Deprivation and Genetic Risk Including Interaction Terms

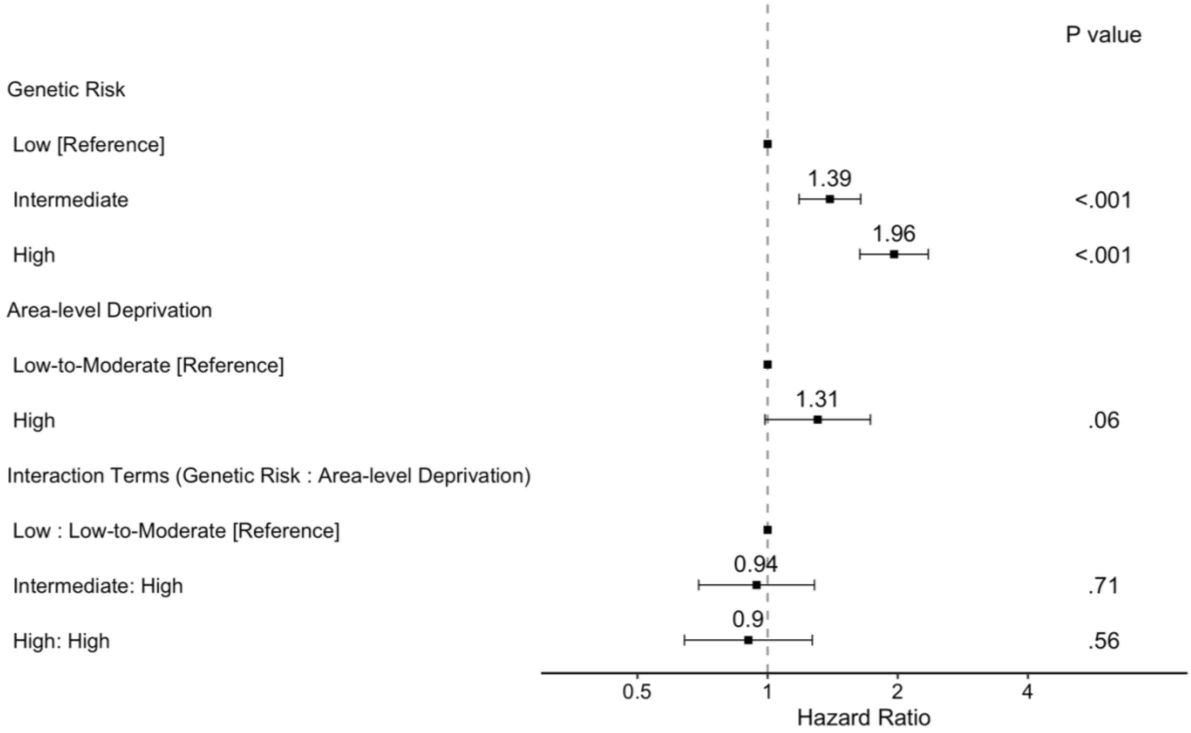

Note. Bars indicate 95% confidence intervals. Hazard ratios are depicted on a log-scale. Colons indicate interaction terms. All Cox proportional-hazards regression model were adjusted for the 20 first PCs, 3rd degree relatedness, number of alleles used to compute the polygenic risk score, age, sex, education, marital status, healthy lifestyle, depressive symptoms in last two weeks and individual-level socioeconomic deprivation.

Appendix Figure 4. Risk of Incident Dementia by Individual-Level Socioeconomic Deprivation and Genetic Risk Including Interaction Terms

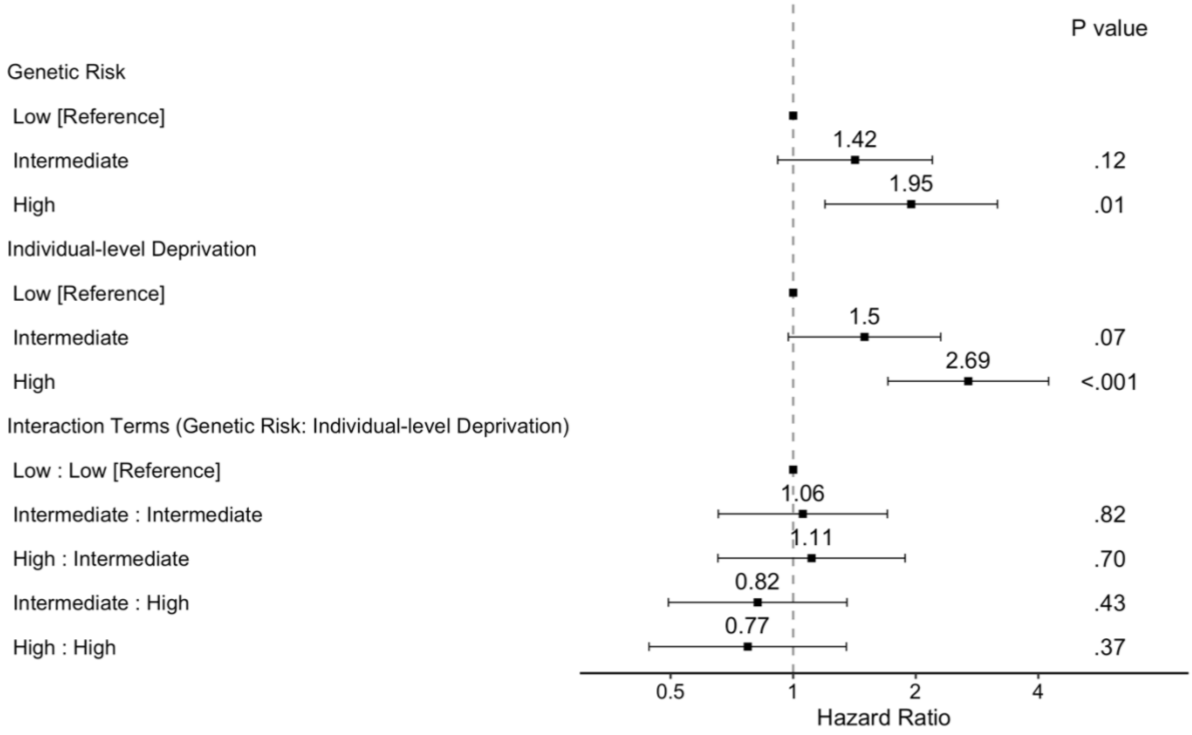

Note. Bars indicate 95% confidence intervals. Hazard ratios are depicted on a log-scale. Colons indicate interaction terms. All Cox proportional-hazards regression model were adjusted for the 20 first PCs, 3rd degree relatedness, number of alleles used to compute the polygenic risk score, age, sex, education, marital status, healthy lifestyle, depressive symptoms in last two weeks and area-level socioeconomic deprivation.
